# Cyclic compressive loading activates angiotensin II type 1 receptor in articular chondrocytes and stimulates hypertrophic differentiation through a G‐protein‐dependent pathway

**DOI:** 10.1002/2211-5463.12438

**Published:** 2018-05-21

**Authors:** Fumihisa Nakamura, Ichiro Tsukamoto, Shinji Inoue, Kazuhiko Hashimoto, Masao Akagi

**Affiliations:** ^1^ Department of Orthopaedic Surgery Kindai University Hospital Osaka‐Sayama Japan

**Keywords:** angiotensin II type 1 receptor, chondrocyte, G‐protein‐dependent pathway, hypertrophic differentiation, mechanosensor

## Abstract

Angiotensin II type 1 receptor (AT1R) appears to have a mechanosensing function in a number of cell types. The purpose of this study was to examine whether AT1R expressed in articular chondrocytes is involved in osteoarthritis (OA) progression *in vivo* and whether cyclic compressive loading activates the AT1R and stimulates hypertrophic differentiation of chondrocytes *in vitro*. The relationships between the modified Mankin score for cartilage degeneration and the expression of AT1R and type X collagen (Col X) were studied in mouse knees with OA induced using the destabilization‐of‐medial‐meniscus model. Cyclic compressive loads were applied to cultured bovine articular chondrocytes in three‐dimensional agarose scaffolds. Expression of Col X and runt‐related transcription factor 2 (Runx2) was analyzed using RT‐PCR and western blotting. We dissected the downstream pathway for intracellular signal transductions of AT1R including G‐protein‐dependent and G‐protein‐independent pathways. Positive significant correlations between the Mankin score and the rate of AT1R‐immunopositive cells and between the rates of AT1R and Col X expression were noted. The expression of Col X and Runx2 was increased by compressive loading but suppressed by addition of olmesartan, an Ang II receptor blocker, to the agarose scaffolds. Compressive loading upregulated the phosphorylation of c‐Jun N‐terminal kinase (JNK), Src, and STAT1, but olmesartan significantly suppressed only JNK phosphorylation. We conclude that AT1R expressed by articular chondrocytes may be involved in OA progression *in vivo*. Mechanical stress can activate AT1R and stimulate hypertrophic differentiation of chondrocytes through the G‐protein‐dependent pathway. AT1R has a mechanosensing function in chondrocytes and may be a new therapeutic target in OA.

**Abbreviations**
Ang II: angiotensin II
ARB: Ang II receptor blocker
AT1R: angiotensin II type 1 receptor
Col X: type X collagen
DMM: destabilization of the medial meniscus
GPCR: G‐protein coupling receptor
IL‐1β: interleukin 1β
JNK: c‐Jun N‐terminal kinase
MTP: metatarsophalangeal
OA: osteoarthritis
qRT‐PCR: quantitative RT‐PCR
RAS: renin–angiotensin system
Runx2: runt‐related transcription factor 2
siRNA: short interfering RNA
STAT1: signal transducer and activator of transcription 1
TM: transmembrane domain
TNF‐α: tumor necrosis factor‐α

Renin was first identified in 1898 by Tigerstedt and Bergman 1. The renin–angiotensin system (RAS) has been studied extensively and is known to play an important role in systemic cardiovascular homeostasis 2. A local tissue‐specific RAS has been identified in many organs and is called the local RAS 3. We previously reported that the local RAS components are expressed in hypertrophic chondrocytes of the mouse epiphyseal plate but not in chondrocytes of normal articular cartilage 4. The angiotensin II (Ang II) type 1 receptor (AT1R) has been reported to be expressed in hypertrophic chondrocytes of human articular cartilage 5. *In vitro*, local RAS components are expressed in cultured chondrocytes 5, 6 and are involved in modulation of chondrocyte hypertrophic differentiation 4, 6, 7. Taken together, these findings suggest that the local RAS components in chondrocytes may be involved in cartilage degeneration through the hypertrophic differentiation of chondrocytes. However, there is insufficient evidence to support this hypothesis.

Angiotensin II type 1 receptor is a member of the G‐protein coupling receptor (GPCR) family of proteins and mediates most of the actions of Ang II 8. Interestingly, Zou *et al*. 9 first reported the mechanosensing function of AT1R in cardiomyocytes in 2004. The authors provided convincing evidence for ligand‐independent, mechanically induced AT1R activation, which contributes to the development of cardiac hypertrophy 9. Application of mechanical stress can result in activation of the AT1R in a number of cell types without involvement of the ligand Ang II 8, 10, 11, and AT1R is now considered to be a representative member of the mechanosensitive GPCR family 8. Cell membrane stretching caused by mechanical stresses induces dislocation and anticlockwise rotation of the transmembrane domain (TM) 7 of AT1R toward the agonist‐binding pocket, which results in activation 8. However, it is unknown whether mechanical stress loaded onto chondrocytes can activate the AT1R and whether this activation stimulates the hypertrophic differentiation of articular chondrocytes.

The purposes of this study were to determine whether AT1R in chondrocytes is involved in the progression of osteoarthritis (OA) *in vivo* and whether cyclic compressive loads, the most important mechanical stress on articular chondrocytes, can activate AT1R and stimulate chondrocyte hypertrophic differentiation *in vitro*. The relationships between cartilage degeneration and immunohistochemical expression of AT1R and type X collagen (Col X) were studied using a mouse knee OA model caused by destabilization of the medial meniscus (DMM). We investigated whether *in vitro* cyclic compressive loads on chondrocytes cultured in three‐dimensional (3D) scaffolds can activate the AT1R. The expression of AT1R and markers of hypertrophic differentiation were investigated using RT‐PCR and western blotting analysis. We also dissected the downstream signal transduction of the AT1R including both G‐protein‐dependent and G‐protein‐independent pathways.

## Materials and methods

### Animals

Eight‐week‐old male C57BL/6 mice were purchased from CLEA Japan Inc. (Tokyo, Japan). Metatarsophalangeal (MTP) joints of 10‐month‐old cows were supplied by Hannan‐Chiksan Co., Ltd. (Habikino, Japan). All experiments were conducted according to the guidelines of the Animal Welfare Committee of Kindai University. Approval from the Animal Welfare Committee of our university was obtained for this study (approval no: KAME‐24‐039).

### Experimental OA model (DMM method)

Experimental OA was induced in 8‐week‐old male C57BL/6 mice, as previously described 12. The right knees of the mice were subjected to DMM. For the sham‐operated group, incision of the skin and joint capsule and irrigation with saline were performed in the left knee of each mouse.

### Staining of mouse chondrocytes

Mouse knee joints were resected 4 and 8 weeks after the operation (*n* = 24) and decalcified as previously described 4. Then, the frontal sliced sections with full‐length anterior crossing ligament were selected and then stained with Safranin O and Fast green; the medial compartments were evaluated using the modified Mankin score 13.

Some series sections were stained immunohistochemically. The expression of AT1R and Col X was evaluated. The immunohistochemical staining was performed as previously described 4. The primary antibodies used were as follows: anti‐AT1R goat polyclonal antibody (1 : 200 in PBS; Santa Cruz Biotechnology, Santa Cruz, CA, USA, #31181) and anti‐Col X rabbit polyclonal antibody (1 : 2000 in PBS; EMD Millipore, Billerica, MA, USA, #234196). Horseradish peroxidase‐conjugated bovine anti‐goat IgG antibody (1 : 1000 in PBS; Santa Cruz Biotechnology, #2350) was used as the secondary antibody for AT1R staining. Horseradish peroxidase‐conjugated bovine anti‐rabbit IgG antibody (1 : 1000 in PBS; Santa Cruz Biotechnology, #2370) was used as the secondary antibody for Col X staining. These stained samples were observed with a light microscope (BZ‐9000; Keyence, Osaka, Japan).

For articular cartilage, the immunohistochemical positive cell‐staining rate was calculated as the number of stained cells relative to the number of chondrocytes in the medial tibial plateau and the medial femoral condyle. First, the number of all chondrocytes contained in the both femoral and tibial articular cartilages of the medial compartment of the knee joint at the frontal sliced sections with full‐length anterior crossing ligament was counted; second, the number of immunohistochemically positive chondrocytes contained in the both the femoral and the tibial articular cartilages of the medial compartment of the knee joint at the same slice was counted. The cells were counted at 200× magnification on an optical microscope. Additionally, the intensity of the staining in the immunohistochemically positive cells in the both femoral and tibial articular cartilages of the medial compartment of the knee joint at the frontal sliced sections with full‐length anterior crossing ligament was also evaluated using the modified Bern score that is a semi‐quantitative scoring system for immunohistochemical staining in chondrocytes (Table 1)^14^, ^15^. The evaluations were performed at 200× magnification on an optical microscope. To investigate whether the expression of the AT1R and Col X is promoted as articular degeneration progresses, correlations between the Mankin score and the immunochemical expression rates of the AT1R and Col X were examined. To investigate the relationship between the expression of the AT1R and Col X, the correlation of immunohistochemical expression rates of AT1R and Col X was examined and the relationship between the Mankin score and the positive cell‐staining rate of Col X relative to AT1R was also calculated.

**Table 1:** The modified Bern score

Uniformity and darkness of immunohistochemical staining in chondrocytes	Score
No stain	0
Weak staining	1
Moderately even staining	2
Even dark staining	3

### Cell culture

**Figure 1:**
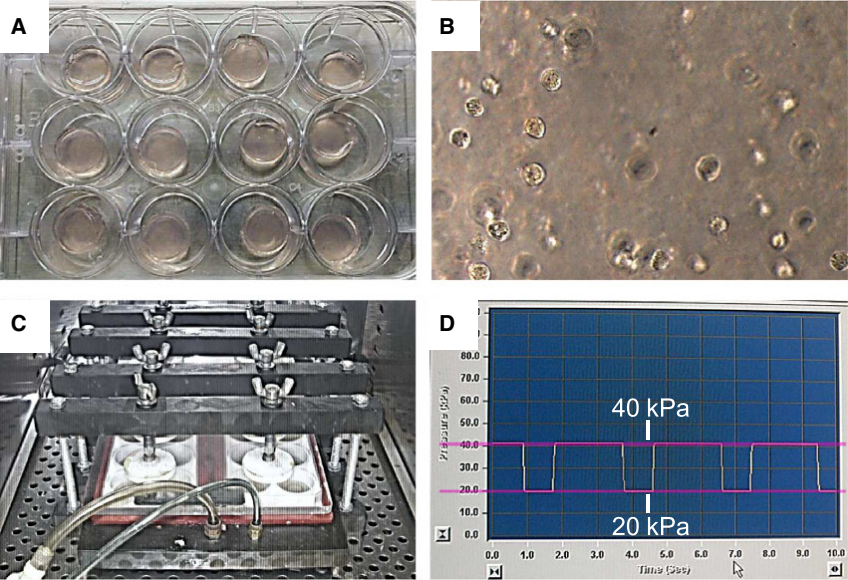
Outline of the cyclic compressive load imposed on the cell–agarose constructs. (A) The cell–agarose construct was formed as a cylindrical plug with a 13 mm diameter and 3 mm height in a 24‐well culture plate. (B) A microscopic photograph of the cell–agarose constructs seeded at a chondrocyte density of 2 × 10^6^ cells·mL^−1^. Magnification: ×200. (C) The compression chambers set in an incubator. (D) The control window of the FX‐4000C (Flexer‐Cell Compression Plus System) indicating that the cyclic compressive load comprised pulses of 20 kPa (2 s on, 1 s off) superimposed on a 20‐kPa static offset pressure.

Chondrocytes were isolated from articular cartilage of MTP joints of a 10‐month‐old cow by digestion with 0.08% collagenase (Wako Pure Chemical Industries, Osaka, Japan) for 6 h at 37 °C. After filtration, cells were seeded at a density of 2 × 10^4^ cells·mL^−1^ in 100 mm plates and cultured until confluent as previously described 4. The cells were then embedded in agarose hydrogels with a cell density of 2 × 10^6^ cells·mL^−1^, as described previously 16, 17 (Fig. 1A,B). The cell–agarose constructs were maintained in culture for 8 days in DMEM at 37 °C in a humidified hypoxic atmosphere (5% O_2_ and 5% CO_2_).

The cell–agarose constructs were allocated into four groups (groups A–D). The constructs in groups A and B were submitted to cyclic compressive loads. The cell–agarose constructs in group B were treated with 10 μm olmesartan, which is an Ang II receptor blocker (ARB), for 12 h before the load. The constructs in group C were treated with 1 μm Ang II for 30 min without load. The constructs in group D were cultured without the addition of any agents or cyclic compressive loads. Olmesartan was obtained from Daiichi‐Sankyo Co., Ltd. (Tokyo, Japan). Ang II was purchased from Sigma‐Aldrich Co., Ltd. (St. Louis, MO, USA).

### Compression stress protocol

The constructs in groups A and B were submitted to cyclic compressive loads using a FX‐4000C™ Flexer‐cell^®^ Compression Plus™ System (Flexcell^®^ International Corp, Burlington, NC, USA) as previously described 17 (Fig. 1C). The loads comprised pulses of 20 kPa (2 s on, 1 s off) superimposed on a 20‐kPa static offset pressure for 30 min (Fig. 1D).

### Quantitative RT‐PCR (qRT‐PCR)

The constructs were lysed using QG buffer and RLT buffer (QIAGEN, Hilden, Germany) as previously described 17. Total RNA was extracted from the solutions using an RNeasy Mini Kit (QIAGEN). The constructs to which agents were administered or to which the cyclic compressive load was applied were treated with the buffers 6 h after administration of the load. The qRT‐PCR analysis was performed as previously described 4. The primer pairs are shown in Table 2.

**Table 2:** The primer pairs used in the quantitative RT‐PCR

	Forward primer	Reverse primer
Col X	5′‐TTCTTCATCCCGTATGCCA‐3′	5′‐GGTGGACCAGGGATGCC‐3′
Runx 2	5′‐ACTGGCGCTGCAACAAGAC‐3′	5′‐CAGTCCCATCTGGTACCTCTCC‐3′
β‐actin	5′‐GGTCATCACCATTGGCAATG‐3′	5′‐CCACAGGACTCCATGCCC‐3′

### Protein extraction

Protein extraction was performed as previously described 17. The exudates were then used for the Bio‐Plex and western blot analyses.

### Western blot analysis

The exudates were adjusted to a protein concentration of 250 μg·mL^−1^ and subjected to polyacrylamide gel electrophoresis in the presence of SDS followed by electrotransfer onto polyvinylidene difluoride membranes (Hybond‐P; Amersham Pharmacia Biotech, Buckinghamshire, UK). The constructs in groups A and B were extracted 48 h after application of the compressive load and in group D after standing for 48 h. The membranes were blocked overnight with Block Ace (Dainippon Sumitomo Pharma, Osaka, Japan) for 1 h and treated with a primary antibody overnight at 4 °C. The primary antibodies were anti‐β‐actin rabbit polyclonal antibody (1 : 1000 in PBS; Cell Signaling Technology, Danvers, MA, USA, #4967), anti‐Col X rabbit polyclonal antibody (1 : 500 in PBS; EMD Millipore, #234196), and anti‐AT1R goat polyclonal antibody (1 : 200 in PBS; Santa Cruz Biotechnology, #31181). The membranes were then treated with horseradish peroxidase‐conjugated secondary antibodies for 1 h while being shaken gently at room temperature. The secondary antibodies used were anti‐rabbit‐IgG horseradish peroxidase‐conjugated goat antibody (1 : 5000 in PBS; Santa Cruz Biotechnology, #2004) for β‐actin and Col X detection, and anti‐goat‐IgG horseradish peroxidase‐conjugated goat antibody (1 : 5000 in PBS; Santa Cruz Biotechnology, #2020) for AT1R detection. Detection was realized using ECL Plus western blotting detection system (Amersham Pharmacia Biotech) and an LAS 4000 charge‐coupled device‐based chemiluminescent analyzer (GE Healthcare Ltd., Buckinghamshire, UK).

### Bio‐Plex analyses

The phosphorylation status of specific signal transduction proteins was analyzed using the Bio‐Plex bead suspension system 18 (Bio‐Rad, Hercules, CA, USA). The constructs in groups A and B were extracted 15 min after the cyclic compressive load and in group C 15 min after the administration of Ang II for 30 min without loading. The exudates were adjusted to a protein concentration of 250 μg·mL^−1^, and the phosphorylation status of three phosphorylated proteins [phosphorylated c‐Jun N‐terminal kinase (JNK), phosphorylated Src, and phosphorylated signal transducer and activator of transcription 1 (STAT1)] was analyzed using Bio‐Plex Pro Cell Signaling Assay Kits (Bio‐Rad).

### Statistical analyses

All *in vitro* experiments were performed six times. Welch's *t* test was used to compare groups. Pearson's correlation test was performed to analyze the relationships between the Mankin score and the positive cell‐staining rates and between the positive cell‐staining rates of Col X and the AT1R. A *P* value < 0.05 was considered significant. The results are presented as mean ± standard deviation and were processed using Microsoft Excel 2010 (Microsoft Corp., Redmond, WA, USA) and a statistical calculating add‐in (Statcel 4; OMS Ltd, Saitama, Japan).

## Results

### *In vivo* analyses

**Figure 2:**
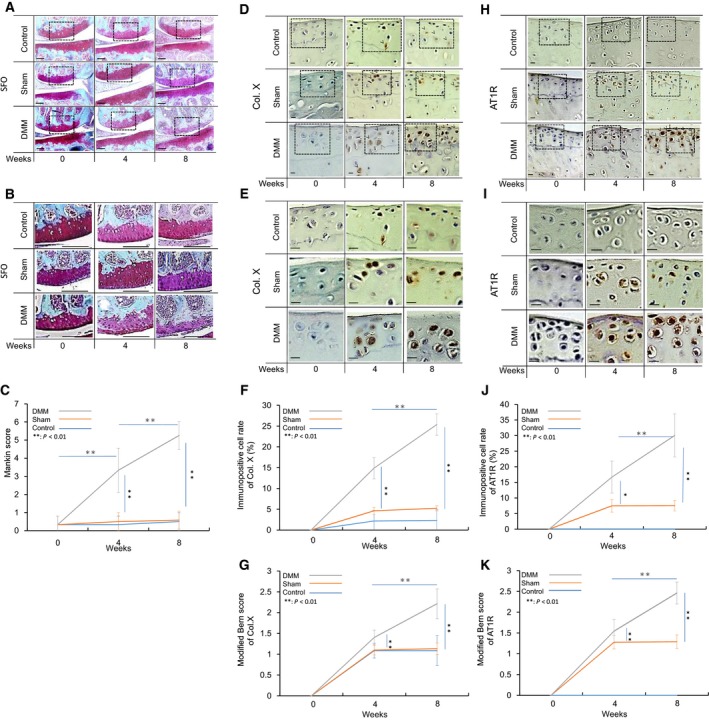
A&B (frontal section of the medial joint compartment), D&E (medial tibial articular cartilage), and H&I (same as D&E) show microscopic photographs of double‐staining with Safranin O and Fast green, and immunohistochemical staining of Col X and AT1R in the articular cartilage of the knee, respectively. Graphs C, F&J, and G&K show the chronological changes in the mean Mankin score, the mean Col X‐ and AT1R‐immunopositive rate, and the mean Bern score, respectively. Dotted squares shown in A, D, and H are the areas for higher magnification views shown in B, E, and I, respectively. Scale bars, A and B: 100 μm; D, E, H, and I: 10 μm. SFO, safranin O. *P < 0.05; **P < 0.01.

In the control group, the Mankin scores were 0.33 ± 0.47, 0.33 ± 0.47, and 0.50 ± 0.50 (*n* = 24) at 0, 4, and 8 weeks, respectively. In the sham group, the scores were 0.33 ± 0.47, 0.50 ± 0.50, and 0.58 ± 0.49 at 0, 4, and 8 weeks, respectively. In the DMM‐operated group, the scores were 0.33 ± 0.47, 3.33 ± 1.21, and 5.25 ± 0.78 at 0, 4, and 8 weeks, respectively. The mean Mankin scores were significantly higher in the DMM mice than in the control and sham‐operated groups at 4 and 8 weeks: *P* = 0.041 and *P* < 0.001 at 4 and 8 weeks, respectively, vs the control and *P* = 0.0052 and *P* < 0.001 at 4 and 8 weeks, respectively, vs the sham‐operated group (*n* = 24 in each group) (Fig. 2A–C).

No Col X‐immunopositive cells were detected at 0 weeks in all groups. The mean ± SD Col X‐positive rates in the control group were 2.17 ± 0.36% (*n* = 24) and 2.31 ± 0.49% at 4 and 8 weeks, respectively. The mean Col X‐positive rates in the sham‐operated group were 4.62 ± 0.82 and 5.22 ± 0.60 at 4 and 8 weeks, respectively. The mean rates of Col X expression in the DMM‐operated group were 14.87 ± 2.57% and 25.36 ± 2.58% at 4 and 8 weeks, respectively. The mean‐modified Col X‐Bern scores in the sham‐operated group were 1.10 ± 0.11 and 1.13 ± 0.14 at 4 and 8 weeks, respectively. The mean‐modified Col X‐Bern scores in the DMM‐operated group were 1.41 ± 0.17 and 2.21 ± 0.36 at 4 and 8 weeks, respectively (Fig. 2D–G).

No AT1R‐immunopositive cells were detected in the control group at 0, 4, or 8 weeks. The mean AT1‐positive rates in the sham‐operated group were 7.49 ± 2.02% and 7.56 ± 1.69% (*n* = 24) at 4 and 8 weeks, respectively. The mean AT1R‐positive rates in the DMM‐operated group were 16.70 ± 5.11% and 30.05 ± 6.90% at 4 and 8 weeks, respectively. The mean‐modified AT1R‐Bern scores in the sham‐operated group were 1.28 ± 0.16 and 1.29 ± 0.16 at 4 and 8 weeks, respectively. The mean‐modified Col X‐Bern scores in the DMM‐operated group were 1.54 ± 0.28 and 2.46 ± 0.27 at 4 and 8 weeks, respectively (Fig. 2H–K).

**Figure 3:**
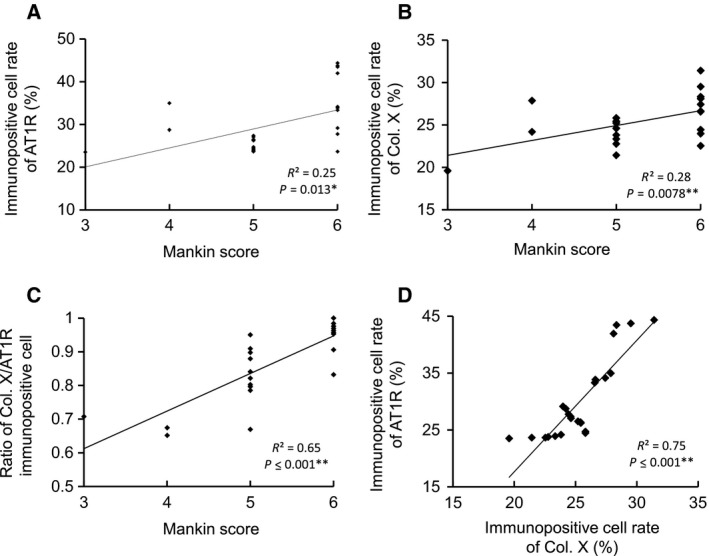
Relationships between the Mankin scores and AT1R‐immunopositive rates (A, *r* ^2^ = 0.25, *P* = 0.013, *n* = 24), between the Mankin scores and Col X‐immunopositive rates (B, *r* ^2^ = 0.28, *P* = 0.0078, *n* = 24), between the Mankin score and ratio of Col X/AT1‐immunopositive rates (C, *r* ^2^ = 0.65, *P* < 0.001, *n* = 24), and between the Col X‐ and AT1R‐immunopositive rates (D, *r* ^2^ = 0.75, *P* < 0.001, *n* = 24). There was a significant relationship between the Mankin score and the ratio of Col X/AT1R‐immunopositive rates, which was always < 1.0. The immunopositive cells were scored and counted in the DMM mice 8 weeks after the index operation. AT1R, angiotensin II type 1 receptor. *P < 0.05; **P < 0.01.

Pearson's correlational analysis showed the following significant positive correlations: (a) between the Mankin score and the AT1R‐immunopositive positive rate in the DMM‐operated group at 8 weeks (*r* ^2^ = 0.25, *P* = 0.013, *n* = 24; Fig. 3A); (b) between the Mankin score and the Col X‐positive rate in the DMM‐operated group at 8 weeks (*r* ^2^ = 0.28, *P* = 0.0078, *n* = 24; Fig. 3B); (c) between the Mankin score and the Col X/AT1R‐positive rate in the DMM‐operated group at 8 weeks (*r* ^2^ = 0.65, *P* < 0.001, *n* = 24; Fig. 3C); and (d) between the Col X‐ and AT1R‐positive rates in the DMM‐operated group at 8 weeks (*r* ^2^ = 0.75, *P* < 0.001, *n* = 24; Fig. 3D).

### *In vitro* analyses

**Figure 4:**
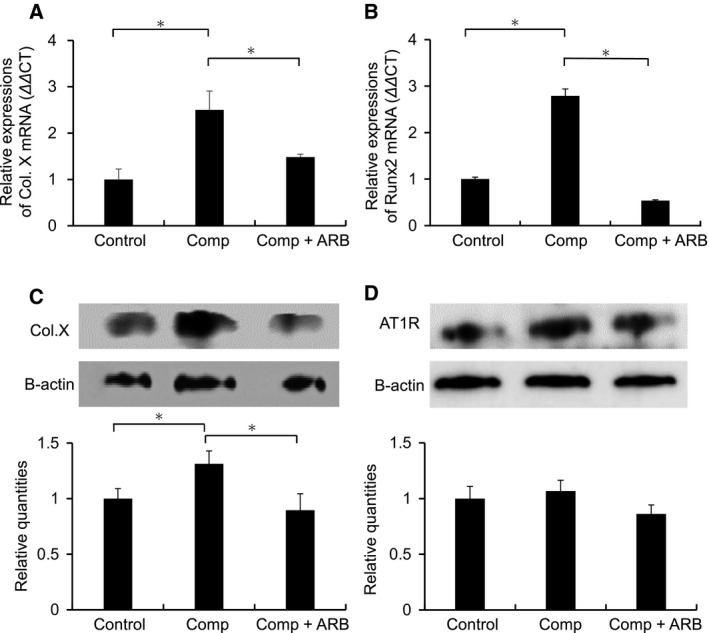
Changes in mRNA expression of Col X and Runx2, and protein expression of Col X and AT1R after application of the cyclic compressive loads. Col X and Runx2 mRNA expression was observed in the control. The mean expression levels of Col X and Runx2 mRNA were significantly upregulated after application of the loads, and this upregulation was reversed by addition of angiotensin II receptor blocker (ARB) olmesartan (A, B). The mean expression level of Col X protein was significantly upregulated after application of the loads, and this upregulation was suppressed by addition of olmesartan (C). Expression of AT1R protein was observed in control chondrocytes. The mean expression level of AT1R protein was not changed by application of the loads or by addition of olmesartan (D). Comp, compression. *P < 0.05.

The mean expression levels of Col X and runt‐related transcription factor 2 (Runx2) mRNA were significantly upregulated after application of the cyclic compressive loads (*P* = 0.023 and 0.033, respectively, *n* = 6; Fig. 4A,B). This upregulation was reversed by the addition of olmesartan to the agarose gel (*P* = 0.017 and 0.016, respectively, *n* = 6; Fig. 4A,B).

The mean expression level of Col X protein was significantly upregulated after application of the loads (*P* = 0.042, *n* = 6; Fig. 4C), and this upregulation was suppressed by addition of olmesartan to the agarose gel (*P* = 0.038, *n* = 6; Fig. 4C). Expression of AT1R protein was noted in control chondrocytes. The mean expression level of AT1R protein was neither changed by application of the loads nor by addition of olmesartan (*P* = 0.44 and 0.085, respectively, *n* = 6; Fig. 4D).

**Figure 5:**
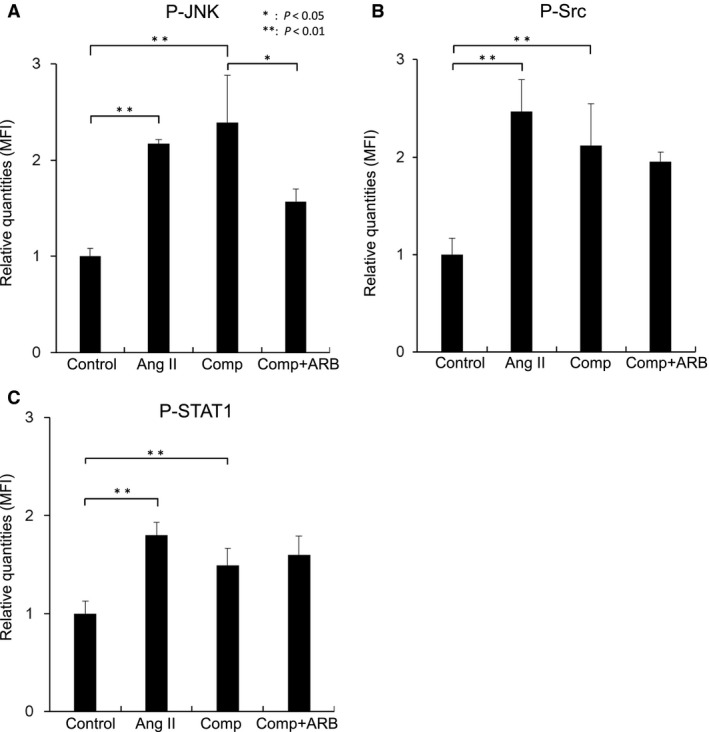
Changes in phosphorylation of signal transduction proteins downstream of AT1R. Addition of Ang II to the agarose gel and application of the cyclic compressive loads significantly upregulated the phosphorylation of JNK. The upregulation of JNK phosphorylation in response to loading was significantly downregulated by the addition of angiotensin II receptor blocker (ARB) olmesartan (A). Addition of Ang II and application of the loads significantly upregulated the phosphorylation of Src and STAT1 (B, C). The upregulation of Src and STAT1 phosphorylation in response to loading was not downregulated by addition of olmesartan (B, C). Comp, compression; MFI, median fluorescence intensity. *P < 0.05; **P < 0.01.

Addition of Ang II to the agarose gel and application of the cyclic compressive loads significantly increased the phosphorylation of JNK compared with the control (*P* = 0.0011 and 0.028, respectively, *n* = 6; Fig. 5A). The increase in JNK phosphorylation caused by the application of the load was significantly attenuated by the addition of olmesartan (*P* = 0.041, *n* = 6; Fig. 5A). Addition of Ang II and application of the load significantly increased the phosphorylation of Src and STAT1 compared with the control (for Src: *P* = 0.00014 and 0.015, respectively; for STAT1: *P* = 0.00026 and 0.0080, respectively, *n* = 6 in each group; Fig. 5B,C). The increase in Src and STAT1 phosphorylation caused by the load was not attenuated by the addition of olmesartan (*P* = 0.57 and *P* = 0.47, respectively, *n* = 6; Fig. 5B,C).

## Discussion

**Figure 6:**
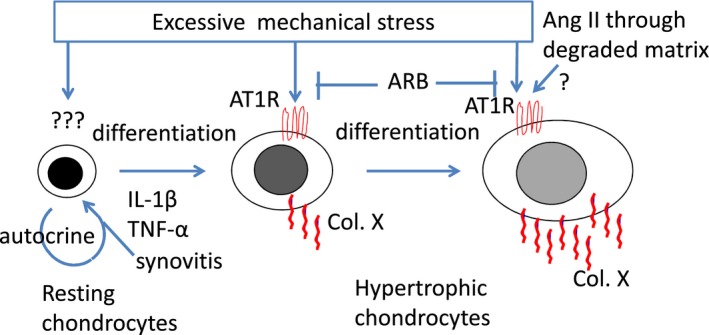
A scheme summarizing the results of this study and the proposed relevance of the mechanosensing function of the AT1R in chondrocyte differentiation. Excessive mechanical stress transmits signals intracellularly through unknown mechanisms, which stimulate hypertrophic differentiation. Inflammatory cytokines such as IL‐1β produced by chondrocytes and/or synoviocytes may increase AT1R expression in chondrocytes. Then, mechanical stress may activate the AT1R to stimulate hypertrophic differentiation further through a G‐protein‐dependent pathway. The mechanical stress and the ligand Ang II, which may access chondrocytes through the degraded cartilage matrix, may synergistically affect the activation of the AT1R to stimulate the hypertrophic differentiation of chondrocytes.

Although a number of mechanosensing mechanisms affecting chondrocyte metabolism have been proposed, including cell adhesion molecules, ion channels, cytoskeletons, and cell surface receptors 19, 20, 21, there is insufficient evidence to show how chondrocytes transmit mechanical stress into intracellular signaling. The results of the present study led us to hypothesize that chondrocytes express the AT1R and that the mechanical load activates the AT1R to stimulate hypertrophic differentiation further through a G‐protein‐dependent pathway (Fig. 6).

Destabilization of the medial meniscus surgery has been reported to accelerate mouse knee OA by increasing the mechanical stresses to articular cartilage 22. It has also been reported that the DMM operation accelerates cartilage degeneration 23 and the expression of Col X 12 in chondrocytes, although the change progresses slowly with age in control and sham‐operated mice 24. Therefore, we have considered that this mode is appropriate to investigate the cartilage degeneration with chondrocyte hypertrophy caused by excessive mechanical stress. Indeed in the present study, the DMM mice showed progressive degeneration of articular cartilage with time after surgery, and both the mean Mankin score and the expression of Col X were significantly higher in the DMM mice than in the control and sham‐operated mice. Additionally, Kawakami *et al*. 5 have reported that AT1R is expressed on the cultured human OA cartilage chondrocyte and that the AT1R expression is enhanced by hypertrophic stimulation with IL‐1. In our previous study, it has been also reported that inflammatory cytokines such as tumor necrosis factor‐α (TNF‐α) and interleukin 1β (IL‐1β) induced the expression of the AT1R in a dose‐dependent manner in primary cultured chondrocytes 4. In the present study, although AT1R and Col X expression was not noted in cartilage at 0 weeks, the immunopositive rates and the immunohistochemical intensity of AT1R and Col X increased significantly in the DMM mice up to 8 weeks after surgery compared with the control and sham‐operated mice. These observations suggest that AT1R expression may be involved in cartilage degeneration caused by the excessive mechanical stress in DMM‐operated knees.

In our previous study, it has been reported that inflammatory cytokines induced the expression of the AT1R in a dose‐dependent manner in primary cultured chondrocytes 4. A certain amount of AT1R expression was noted in sham‐operated mice at 4 and 8 weeks after the operation. Surgical invasion caused by the sham operation may have stimulated the production of these proinflammatory cytokines. The expression level of AT1R increased significantly from 4 to 8 weeks in the DMM mice but did not change over the time period in the sham‐operated mice; the levels were significantly higher at 4 and 8 weeks in the DMM mice than in the sham‐operated mice. These results suggest that the interplay between mechanical, inflammatory, and biochemical factors may affect AT1R expression in cartilage under normal or pathological conditions. The increase in AT1R expression over time in the DMM mice may be attributable to the additive or synergistic effects of these factors, which may interact with each other during OA progression.

A significant relationship between the Mankin score and AT1R‐immunopositive rate in chondrocytes and a strong relationship between AT1R‐ and Col X‐immunopositive rates in chondrocytes in degenerated cartilage were noted. A positive significant relationship was observed between the Mankin score and the ratio of Col X/AT1R‐immunopositive cells, which was always < 1.0. These results suggest that there is a cause‐and‐effect relationship between the expressions of these two proteins.

Additionally, although AT1R expression was not noted in cartilage at 0 weeks in mice, expression of AT1R protein in the control cultured bovine chondrocytes, which was not subjected to compressive load, was noted. This discrepancy suggests the probability of the presence of very low levels of AT1R protein in the resting chondrocytes of mice that cannot be detected by immunohistochemical staining.

To study whether the AT1R expressed in chondrocytes can transduce mechanical stress to intracellular signals and thereby stimulate hypertrophic differentiation, *in vitro* experiments were performed. First, it had been confirmed that AT1R expression occurred with Col X expression in chondrocytes cultured in 3D agarose scaffolds for 8 days (Control in Fig. 5C,D), and then, cyclic compressive loads were applied on these cells using the FX‐4000 system 17. The protocol for the cyclic compressive loads used in the present study is known to decrease the promoter activity of the Col II gene in 3D‐cultured chondrocytes 16, 25. This suggests that the load applied to the chondrocytes in the agarose gel may have provided excessive mechanical stress on the chondrocytes even though the maximum load to the agarose gel was 40 kPa. However, the cyclic compressive loads used in this study upregulated the expression of Runx2 and Col X mRNA, and the addition of the olmesartan to the gel significantly attenuated this upregulation. The protein expression of Col X was also upregulated by the cyclic loads and was significantly attenuated by olmesartan. These results suggest that the loading activated the AT1R in chondrocytes independent of the ligand and stimulated hypertrophic differentiation through activation of the AT1R because, in other cell types, olmesartan can block the mechanically induced activation of the AT1R by binding to the physiologically active sites of the AT1R, as described in the following 11.

In the present study, olmesartan, a known potent ARB with inverse agonist activitiy 26, 27 similar to that of candesartan 8, was used as an AT1R inhibitor. The carboxyl domain of candesartan binds to Gln257 in TM6 and to Thr287 in TM7, and these interactions can constrain two TM domains until the receptor is stabilized to the inactive state, through which this ARB can exhibit inverse agonist activity 28. The suppressive effects of olmesartan on the mechanosensing functions of AT1R shown in the present study may be attributed to the inverse agonist activities of this ARB.

Finally, the downstream signaling pathways of the AT1R that were activated by the cyclic compressive loading, including G‐protein‐dependent and G‐protein‐independent pathways, were dissected. Interestingly, it has been reported that ligand‐independent mechanical stress activates some cell surface molecules such as integrins, which are TM receptors that facilitate extracellular matrix binding and have Src in their downstream pathway 29, and vascular endothelial growth factor receptor 2, which has STAT1 in its downstream pathway 10, 30. Phosphorylation of signaling proteins including JNK, Src, and STAT1 was detected after application of the loads, which indicates that the loads can lead to the activation of both G‐protein‐dependent and G‐protein‐independent pathways. However, among the signaling proteins upregulated, addition of olmesartan to the agarose gel reversed the phosphorylation of JNK only. Considering that olmesartan could not reverse the upregulation of Src and STAT1 phosphorylation, there could be other mechanosensing pathways in chondrocytes except the pathway through AT1R, which may have the Src and STAT1 in their downstream.

We acknowledge some limitations in the present study. To reveal the clinical relevance of the present study, it is important to verify whether the articular cartilage damages could be improved in the DMM mice by administration of olmesartan and other antihypertensive drugs. Additionally, whether the mechanosensitive effects of AT1R shown in the vitro should effective in actual articular joints is still unknown. In these points, further investigations might be needed. Additionally, we had better consider about the use of genetically modified animals to reveal whether the modification in the expression of the RAS may influence the progression of the OA *in vivo*. Fukamizu *et al*. 31 have reported that they had made Tsukuba hypertensive mice, which have been transfected the human angiotensinogen gene and human renin gene resulting in overexpression of Ang II in whole body. Tanimoto *et al*. 32 also have reported that they had made angiotensinogen‐deficient mice with hypotension. It might be useful to use these genetically modified mice for investigating the relation between the RAS and the progression of OA. In addition to the administration of the ARB, it might be very important to perform compressive load experiments with chondrocytes silenced for AT1R. The short interfering RNA (siRNA) oligonucleotides method is usually effective in gene silencing. In preliminary experiment for the present study, the transient transfection of the siRNA oligonucleotides for AT1R was performed to the cells, which were at about 30% confluent, using Oligofectamine (Thermo Fisher Scientific, Waltham, MA, USA) according to the manufacturer's protocols resulting in a final siRNA concentration of 200 nm added to the cells. After culturing to the confluent for about 5 days, the cells were then embedded in agarose hydrogels and were maintained in culture for 8 days; then, the protein extraction and the following western blot analysis for AT1R were also performed as in same methods as the examination in the present study. However, knockdown or downregulation of AT1R was not detected; the data are not shown. The period between the transfection and the detection, which was almost 13 days, might be long. To the best of our knowledge, the period, in which transfected siRNA is effective, is only several days. To obtain the long‐term knockdown effect, short hairpin RNA technique with lentiviral vector might be effective. However, this method includes some intricate high technique. Unfortunately, our cell biological technique has not reached this level. The further compressive load experiments with chondrocytes silenced for AT1R have been still needed. Additionally, to reveal how dominant the mechanosensing functions of AT1R, which is show in the present study, is in the progression of actual human OA, further *in vitro* studies using human articular chondrocytes from OA patients should be needed. Previously, Kawakami *et al*. 5 have reported that AT1R is expressed on the cultured human OA cartilage chondrocyte and that the AT1R expression is enhanced by stimulation with IL‐1; they also have mentioned ‘Chondrocytes from all patient types expressed AT1R/AT2R mRNA, though considerable variation was found between samples’. We have same concern as that of Kawakami *et al*.'s. In performing total knee or hip arthroplasties, it has been almost always observed that degrees of the degeneration of the articular cartilage are really various for each portion even in the same joint. Considering the variety, the further study investigating the mechanosensing functions of AT1R in actual human OA articular cartilage chondrocytes should be considered according to their each degrees of degeneration.

In conclusion, mechanical stress can activate the AT1R and stimulate hypertrophic differentiation through a G‐protein‐dependent pathway. The AT1R has a mechanosensing function in chondrocytes and thus may be a new therapeutic target in the treatment of OA. Further studies are needed to elucidate whether the AT1R works as a mechanosensor in physiological metabolism and/or repair processes in cartilage and whether the mechanical stresses and the ligand Ang II synergistically affect the activation of the AT1R to stimulate hypertrophic differentiation of chondrocytes (Fig. 6).

## Author contributions

All authors conceived and designed the project; all authors acquired the data; all authors analyzed and interpreted the data; all authors wrote the study.
